# Monotreme-specific conserved putative proteins derived from retroviral reverse transcriptase

**DOI:** 10.1093/ve/veac084

**Published:** 2022-09-03

**Authors:** Koichi Kitao, Takayuki Miyazawa, So Nakagawa

**Affiliations:** Laboratory of Virus-Host Coevolution, Institute for Life and Medical Sciences, Kyoto University, 53 Kawahara-cho, Shogoin, Sakyo-ku, Kyoto 606-8507, Japan; Laboratory of Virus-Host Coevolution, Institute for Life and Medical Sciences, Kyoto University, 53 Kawahara-cho, Shogoin, Sakyo-ku, Kyoto 606-8507, Japan; Department of Molecular Life Science, Tokai University School of Medicine, 143 Shimokasuya, Isehara, Kanagawa 259-1193, Japan

**Keywords:** endogenous retrovirus, monotreme, reverse transcriptase, virus-derived gene

## Abstract

Endogenous retroviruses (ERVs) have played an essential role in the evolution of mammals. ERV-derived genes are reported in the therians, many of which are involved in placental development; however, the contribution of the ERV-derived genes in monotremes, which are oviparous mammals, remains to be uncovered. Here, we conducted a comprehensive search for possible ERV-derived genes in platypus and echidna genomes and identified three reverse transcriptase-like genes named *RTOM1*, *RTOM2*, and *RTOM3* clustered in the *GRIP2* intron. Comparative genomic analyses revealed that *RTOM1*, *RTOM2*, and *RTOM3* are strongly conserved and are under purifying selection between these species. These could be generated by tandem duplications before the divergence of platypus and echidna. All *RTOM* transcripts were specifically expressed in the testis, possibly suggesting their physiological importance. This is the first study reporting monotreme-specific *de novo* gene candidates derived from ERVs, which provides new insights into the unique evolution of monotremes.

## Introduction

Endogenous retroviruses (ERVs) are remnants of retroviral genomes found in the host genomes. ERVs are retroviruses that infected the host germline cells and were integrated into the host genome (Johnson 2019). Young ERVs retain their viral open reading frames (ORFs) but gradually lose their intact ORFs due to the accumulation of mutations. However, proteins expressed from ERVs sometimes evolve as functional genes in the host (Ueda et al. 2020). A typical example is the syncytin genes, ERV-derived fusogenic genes, which are expressed in the human placenta (Blaise et al. 2003; Blond et al. 2000; Mi et al. 2000) and are essentially required for mouse placenta formation (Dupressoir et al. 2009, 2011). Syncytin genes have been independently acquired from different ERVs in different mammalian lineages, which is a representative example of the convergent evolution (Imakawa, Nakagawa, and Miyazawa 2015). In addition, other ERV-derived genes that do not show fusogenic activity have also been found to be expressed in the placenta. For example, *HEMO* encoding a secreted envelope protein (Heidmann et al. 2017) as well as *gagV1* and *pre-gagV1* genes (Boso et al. 2021) are highly expressed in the human placenta. Restriction factors against exogenous retroviruses are another example of viral gene co-option. For example, *gag*-derived *Fv1* (Best et al. 1996) and *env*-derived *Fv4* (Ikeda and Sugimura 1989) inhibit retroviral infection in mice. Despite these contributions to the evolution of therians, it is still unclear whether ERV-derived genes are co-opted in monotremes (egg-laying mammals).

Here, we attempted to determine whether there are ERV-derived genes specific to monotremes. Comparative studies for the detection of ERV-derived genes have been conducted in mammalian genomes, including the platypus (Nakagawa and Takahashi 2016; Wang and Han 2020). However, for monotremes, only the genome sequence of one species, the platypus, was available (OANA5), the quality of which was limited (Warren et al. 2008). Recently, high-quality monotreme genomes of platypus (mOrnAna1.p.v1) and echidna (mTacAcu1.pri) were determined using long-read sequencing technology (Zhou et al. 2021). Taking advantage of these genome sequences, we conducted comparative analyses and detected three novel ERV-derived genes specific to the monotreme lineage.

## Results and discussion

**Figure 1.**
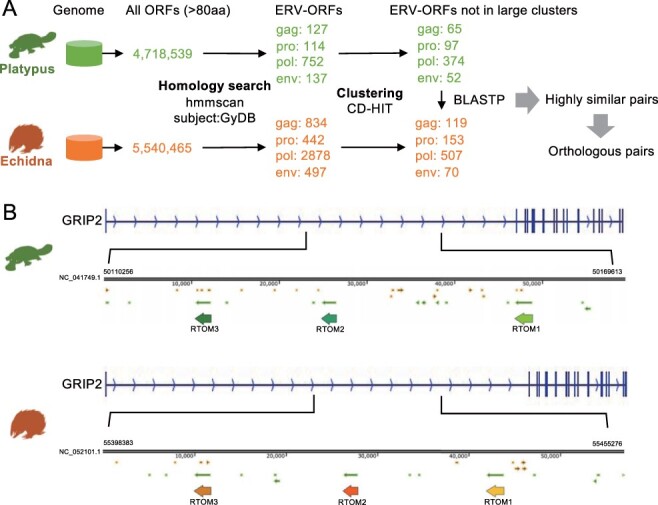
Identification of *RTOM1*, *RTOM2*, and *RTOM3*. (A) Schematic representation of the in silico screening for conserved ERV-derived genes in platypus and echidna. (B) Genomic context of *RTOM1*, *RTOM2*, and *RTOM3*. The thin arrows indicate ORFs above 100 amino acid length. The nucleotide sequences of *RTOM* ORFs are available in Supplementary File 2.

To comprehensively search for ERV-derived genes in the genomes of monotremes, we extracted ORFs from the genomes of platypus and echidna. The amino acid sequences obtained by the virtual translation of these ORFs were used as queries for the sequence search. We used the hidden Markov model (HMM) of retroviral genes in the Gypsy Database 2.0 (GyDB) (Llorens et al. 2011) as the subject of the sequence search (Supplementary Table S1). We identified ORFs similar to *gag, pro, pol*, and *env* genes (Fig. 1A). These ORFs are presumed to be a mixture of ORFs that (1) have physiological functions and are evolutionarily conserved and (2) have not been disrupted by nonsense mutations by chance. Most ERV ORFs belonging to (2) are derived from young ERVs, in which mutations have not yet accumulated. To exclude young ERV ORFs, we performed the clustering analysis based on the amino acid sequence identity. Since young ERVs are thought to be included in large clusters due to their mutual similarity, we removed sequences that belonged to large clusters consisting of more than ten sequences. This step could also exclude evolutionarily conserved but highly duplicated genes such as SCAN domain-containing genes (Emerson and Thomas 2011), which is beyond the scope of this study. Next, using the platypus ORFs as queries and the echidna ORFs as the subjects, we conducted a sequence similarity search using BLASTp. We obtained ORF pairs with high amino acid similarity (Supplementary Fig. S1A and Table S2). One of these ORFs was *ASPRV1* that is a known ERV-derived protease gene acquired in the common ancestor of mammals and is responsible for skin maintenance (Matsui et al. 2011). Such ERV-derived ORFs that are annotated as genes in the human genome were removed, and three ORFs remained (Supplementary Table S2). They were located tandemly in the intron of the *GRIP2* gene in the opposite direction (Fig. 1B). All three ORFs showed high similarity to the reverse transcriptase (RT) of spumaretrovirus in GyDB (Supplementary Table S3). Therefore, we designated these gene candidates as *RTOM* [RT-like ORF in Monotreme], and the three were named *RTOM1, RTOM2*, and *RTOM3* in order of their location from the 5′ direction (Fig. 1B). To further examine the genomic loci of the *RTOM* ORFs in monotremes, We performed self-alignment of the GRIP2 gene including the three ORFs for the platypus and echidna genomes using LAST program (Kiełbasa et al. 2011). The dot-plots indicate that the three ORFs, including the surrounding regions, were aligned as tandem repeats (Supplementary Fig. S2). We also attempted to align platypus and echidna *GRIP2* with the human and mouse *GRIP2* to gain more insights into the structural evolution of this region including the *RTOM* ORFs; however, the introns of *GRIP2* were not conserved among them, suggesting that the *RTOM* ORFs could emerge in the ancestor of platypus and echidna (Supplementary Fig. S3).

To examine the possibility that the *RTOM* ORFs were acquired before the divergence of therians and monotremes, the nucleotide and amino acid sequences of the *RTOM* ORFs were searched using BLASTn and tBLASTn, respectively, with an e-value < 1E − 5 against all genomes of mammals, birds, and reptiles available in the National Center for Biotechnology Information (NCBI) Assembly. The BLASTn search resulted in significant hits from several genomes (mammals: 26 out of 510 genomes, birds: 29 out of 556 genomes, and reptiles: eight out of seventy-nine genomes) (Supplementary Table S4); however, their query cover rates were low (up to 5.2 per cent) except for hits to the *RTOM* ORFs themselves. The tBLASTn search identified up to thousands of hits for each genome (Supplementary Table S4). This is because the amino acid sequences of the *RTOM* ORFs are similar to the RT region of other ERVs. We examined the proximity of these hits to the *GRIP2* gene and found no hits considered to be orthologs of the *RTOM* ORFs (see the ‘Materials and Methods’ section). Therefore, we conclude that the *RTOM* ORFs are monotreme-specific.

**Figure 2.**
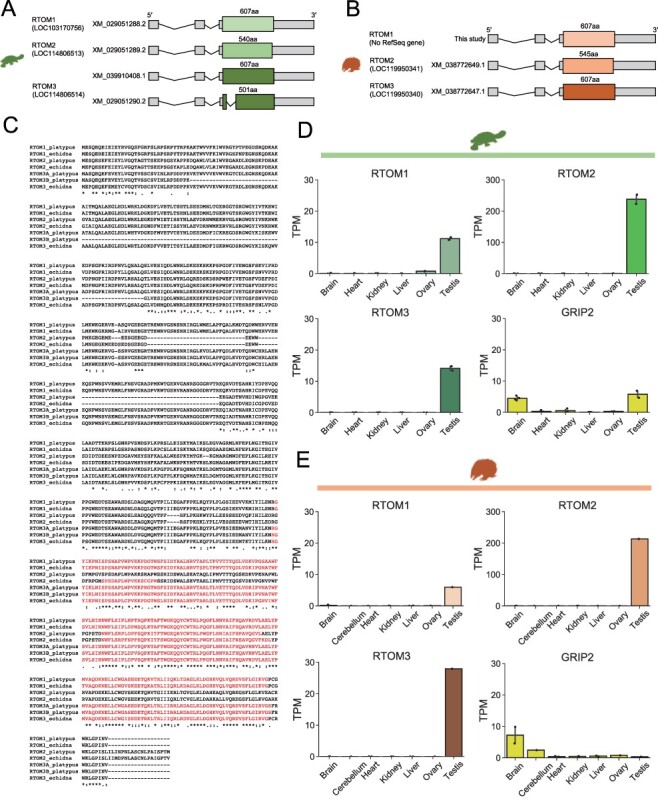
Expression of *RTOM1, RTOM2*, and *RTOM3*. (A) Schematic representation of the RefSeq transcripts of the *RTOM* genes in platypus. (B) Schematic representation of the reconstructed *RTOM1* transcript and RefSeq transcripts of the *RTOM2* and *RTOM3* genes in echidna. (C) Multiple alignment of the amino acid sequences of RTOMs. The amino acid sequence of echidna RTOM1 was obtained from the genomic ORF. ‘RTOM3A_plasypus’ and ‘RTOM3B_platypus’ are isoforms derived from ‘XM_039910408.1’ and ‘XM_029051290.2,’ respectively. The regions showing similarity to the HMM of spumaretrovirus RT domain in GyDB are indicated in red. (D, E) Tissue-specific expression of *RTOM* genes and *GRIP2* in (D) platypus and (E) echidna. Normalized expression levels are presented as transcript per million (TPM).

We found that, in the platypus genome, there are computationally annotated RefSeq genes containing the *RTOM* ORFs (Fig. 2A). *RTOM1*, *RTOM2*, and *RTOM3* genes of platypus contain two introns in the 5′ UTR, and the entire *RTOM* ORFs are expressed as mRNA excluding a second splicing variant of *RTOM3* that partially lost its ORF (Fig. 2A). In echidna, *RTOM2* and *RTOM3* gene structures were annotated in the RefSeq transcripts; however, *RTOM1* was not annotated. By conducting transcriptome assemblies of RNA-seq data of echidna tissues (Supplementary Table S5), we reconstructed all *RTOM* transcripts including the *RTOM1* (Fig. 2B; Supplementary Fig. S4). We also found a chimeric transcript of *RTOM2* and *RTOM3*, which was transcribed from the transcription start site of *RTOM2*, but its CDS is *RTOM3* (Supplementary Fig. S4). Except for this chimeric transcript, all echidna *RTOM* transcripts have two introns in the 5′ UTR, which was similar to those of platypus. We then constructed a multiple alignment of the seven amino acid sequences of platypus and echidna RTOMs, including two splicing variants of platypus *RTOM3* (Fig. 2C). The amino acid sequence of RTOM2 lacks a region shared by RTOM1 and RTOM3, but the C-terminal region was conserved among the amino acid sequences of RTOMs without insertion or deletion (Fig. 2C). To investigate the tissue-specific expression of the *RTOM* genes, we analyzed the RNA-seq data of platypus and echidna (Supplementary Table S5). In platypus, *RTOM1*, *RTOM2*, and *RTOM3* were commonly highly expressed in the testis (Fig. 2D). *GRIP2* was expressed not only in the testis but also in the brain, and its expression level was lower than that of the *RTOM* genes. We further investigated the mapped reads using Interactive Genome Viewer (Thorvaldsdóttir, Robinson, and Mesirov 2013) (Supplementary Fig. S5) and found that *RTOM3* showed a splicing variant with an intron in the coding region, as shown in the RefSeq transcript. In echidna, we found that all *RTOM* transcripts were specifically expressed in the testis, similar to platypus. Expression of *GRIP2* in echidna testis was also relatively low (Fig. 2E). This suggests that the *RTOM* genes are more actively transcribed than *GRIP2*, or the *RTOM* transcripts are more stable than the *GRIP2* transcripts in the testis. Given the higher expression level of *RTOM2* in both platypus and echidna, this gene may play a central role in the *RTOM* genes. It is still possible that the relative expression levels of three genes may change according to other tissues and developmental stages that were not examined in this study. In addition, since this study did not present the evidence of the translation of the *RTOM* transcripts, whether putative RTOM proteins are involved in testicular function needs to be verified in the future.

**Figure 3.**
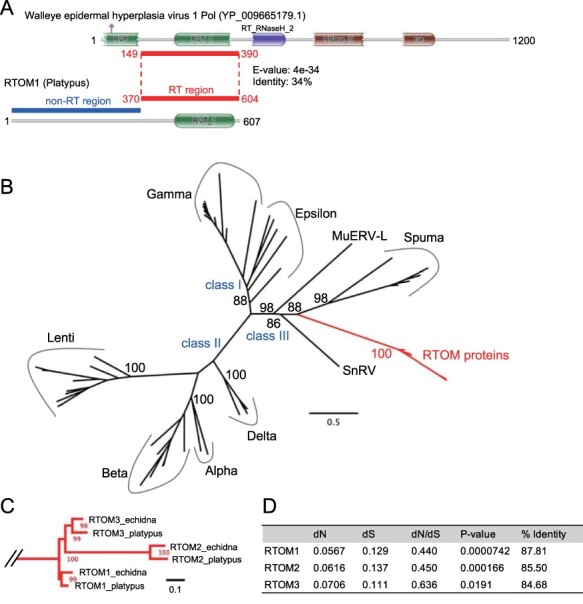
Evolution of *RTOM1*, *RTOM2*, and *RTOM3*. (A) Comparison between platypus RTOM1 and retroviral Pol protein. Walleye epidermal hyperplasia virus 1 is represented as an example. A region showing similarity to the Pol protein by BLASTp was designated as ‘RT region’. A region that did not show similarity to any retroviral genes was designated as ‘non-RT region’. (B) A phylogenetic tree constructed from the amino acid sequences of RT regions of the six RTOMs and the retroviral Pol proteins in GyDB. The multiple alignment is available in Supplementary File 3. Ultrafast-bootstrap values obtained from 1000 times replication are shown in major branches. (C) Detailed representation of the clade of the amino acid sequences of RTOMs. The scale was shown in the right bottom. (D) The numbers of nonsynonymous substitutions (dN) and synonymous substitutions (dS) per site estimated by Nei–Gojobori method (Nei and Gojobori 1986). Statistical significance of selection was estimated by the codon-based Z test of neutrality using MEGA-X (Kumar et al. 2018). The % identity was calculated using the Ident and Sim program (https://www.bioinformatics.org/sms2/ident_sim.html) (Stothard 2000).

To obtain insights into the viral origin of the *RTOM* genes, we performed a BLASTp search of the amino acid sequence of platypus RTOM1 against the NCBI virus database. We found that retrovirus Pol proteins from various distinct lineages, namely gammaretrovirus, deltaretrovirus, epsilonretrovirus, and spumaretrovirus, are similar to the amino acid sequence of RTOM1 (BLASTp: E-value < 1E − 20). In all hits, the retroviral Pol proteins showed high similarity to the latter half of RTOM1 (approximately 370-607aa) (Fig. 3A). A domain search against the Pfam database (Mistry et al. 2021) in the HMMER web service (Finn, Clements, and Eddy 2011) revealed that the latter half of RTOM1 and RTOM3 contain RT domains (Supplementary Fig. S6). A phylogenetic tree was constructed from the RT regions of the amino acid sequences of RTOMs and the retroviral Pol proteins (Fig. 3B). The amino acid sequences of RTOMs appear to be more related to class III retroviruses, including spumaviruses or spumavirus-related MuERV-L (Llorens et al. 2009). The tree topology of the RTOMs strongly suggests that all the *RTOM* genes were formed before the divergence of platypus and echidna. Together with the self-alignment of genomic sequences (Supplementary Fig. S2), three *RTOM* genes were generated by tandem gene duplications before the divergence of platypus and echidna (Fig. 3C). In the non-RT region of the amino acid sequences of RTOM1 (approximately 1–369aa), no significant hits for retroviruses were obtained (Fig. 3A). We performed a BLASTp search for all non-redundant proteins in the GenBank database for the non-RT region of the amino acid sequences of RTOM1; however, no similar proteins were found except for the putative proteins of RTOM2 and RTOM3 (E-value < 0.05). This suggests that the non-RT region was derived from a non-retroviral sequence or was derived from the retroviral gene that has accumulated too many mutations to be aligned with retroviruses. Considering the structural divergence of the non-RT region, such as deletion of RTOM2 and splicing variant of platypus RTOM3 (Fig. 2C), the RT region is a core domain of the putative RTOM proteins, and the non-RT region may provide functional modifications specific to each putative RTOM protein.

During the 187-million-year history of diverging from monotremes, therians have acquired many ERV genes and evolved their unique features, especially the placenta (Imakawa and Nakagawa 2017). Our work revealed that monotremes also domesticated ERV genes that emerged and were conserved more than 55 million years ago, the divergence time of platypus and echidna (Zhou et al. 2021). Although the translation of *RTOM* genes was not confirmed in this study, the calculation of nonsynonymous and synonymous nucleotide substitution frequencies of *RTOM1*, *RTOM2*, and *RTOM3* shows that their amino acid sequences are under purifying selection, strongly suggesting that they physiologically function as proteins (Fig. 3D). We found that the RT domain of all putative RTOM proteins lacked the three catalytic carboxylates of aspartic acids (Supplementary Fig. S7). These amino acid residues are highly conserved among all retroviruses, and the replacement of these amino acids results in a complete loss of the RT activity (Larder et al. 1987; Sarafianos et al. 2009). Therefore, the putative RTOM proteins may have different functions from those of reverse transcription. To the best of our knowledge, there are no retroviral genes in which only the RT domain is co-opted in vertebrates (Naville et al. 2016). Although this study has a limitation that the expression and function of the putative RTOM proteins have not been fully validated due to difficulties in obtaining tissues, the future functional elucidation of *RTOM1*, *RTOM2*, and *RTOM3* will provide us with new aspects of ERV-derived genes functioning in mammals.

## Materials and methods

### Identification of conserved ERV genes

The platypus genome (mOrnAna1.p.v1, GCF_004115215.1) and the echidna genome (mTacAcu1.pri, GCF_015852505.1) were used for the ERV gene screening. The 240-nt ORF flanked by stop codons was retrieved using the getorf program in the European Molecular Biology Open Software Suite (Rice, Longden, and Bleasby 2000). For HMM-based sequence search, hmmscan was used (expected threshold: 1E − 5) in HMMER3 v3.2.2 (Eddy 2011). ORFs were clustered using CD-HIT v4.8.1 (Li and Godzik 2006) with 50 per cent amino acid identity. The sequence search for platypus ORFs against echidna ORFs was conducted using BLASTp v2.10.0+ with an e-value < 1E − 50 (Camacho et al. 2009). By examining the distribution of the bitscore of the BLASTp search, we extracted ORF pairs that showed high similarity between the two species (Supplementary Fig. S1A). Then, we examined the RefSeq annotation and identified the genes to which the ORFs belonged.

To examine the presence of homologous sequences beyond the monotreme lineage, the genes that were not described in the human RefSeq genes were subjected to deep homology searches. We performed BLASTn and tBLASTn v2.10.0+ with e-values < 1E − 5 against all genomes of mammals, birds, and reptiles downloaded on 22 January 2022 (Supplementary Table S4). The query cover rate for each hit was calculated as [alignment length/query length]. To examine whether the amino acid sequences obtained by tBLASTn are the ortholog of the *RTOM* genes, we investigated the proximity of these hits to the *GRIP2* gene as follows. First, we extracted hits to the amino acid sequences of RTOMs with a query cover of at least 60 per cent. Second, to obtain the genomic position of *GRIP2* on each genome, we performed BLAT v0.35 (Kent 2002) using the amino acid sequence of the human GRIP2 (NP_001073892.3) against the 1,145 genomes. We considered the hit with the highest score in each genome as the *GRIP2* position. Finally, we compared the genomic position of the hits of *RTOM* and *GRIP2* and confirmed that all of them were located on different contigs or were located far enough from each other (the closest pair of hits to *RTOM* and *GRIP2* in the same contig is 7.7 Mb apart from each other).

To validate this approach, we performed similar analyses on the genomes of humans (GRCh38.p13) and marmosets (Callithrix_jacchus_cj1700_1.1) (Supplementary Fig. S1B and C). They diverged 43 million years ago (Perelman et al. 2011). We successfully identified known ERV-derived genes such as *PEG10* (Ono et al. 2001), *RTL1*/*PEG11* (Charlier et al. 2001), *ASPRV1* (Matsui et al. 2011), *NYNLIN*/*CGIN1* (Marco and Marín 2009), *ERVV-1* and *2* (Kjeldbjerg et al. 2008), and *ERVMER34-1*/*HEMO* (Heidmann et al. 2017) (Supplementary Table S6). This suggests that our method is sensitive enough to identify ERV ORFs conserved in platypus and echidna.

### Expression analyses

RNA-seq data of platypus (twenty samples from six tissues) (Marin et al. 2017) and echidna (eleven samples from seven tissues) (Zhou et al. 2021) were used (Supplementary Table S5). Low-quality reads were trimmed and filtered using fastp v0.19.5 with default options (Chen et al. 2018). The filtered reads were mapped to each reference genome using HISAT2 v2.1.0 with default option (allowing ≤5 multiple mappings) (Pertea et al. 2016). Based on the eleven RNA-seq sequencing data mapped on the echidna genome, we obtained the echidna *RTOM1* transcript by conducting transcriptome assembly using Stringtie2 v2.1.6 with ‘--merge’ option (Kovaka et al. 2019). We have added the coordinates of the echidna *RTOM1* transcript (Supplementary File 1) to the RefSeq gene coordinates. We then calculated the expression levels for twenty platypus and eleven echidna RNA-seq samples using the Stringtie2 program with default options (Kovaka et al. 2019). To extract unique-mapped reads from a given aligner-generated SAM file, we collected reads containing the ‘NH:i:1’ flag indicating that they were uniquely mapped into the genome.

### Phylogenetic analyses

Representative retroviral Pol amino acid sequences were retrieved from the GyDB collection (https://gydb.org/index.php/Alignment?alignment=POL_retroviridae_Biology_Direct_4_41_2009&format=txt) (Llorens et al. 2009). A multiple alignment was generated using MAFFT v7.487 (Katoh and Standley 2013), and poorly aligned regions were removed using trimAl v1.4.rev15 (Capella-Gutiérrez, Silla-Martínez, and Gabaldón 2009). A phylogenetic tree was constructed using IQ-TREE2 v2.0.8 (Minh et al. 2020) with 1,000 replicates of ultrafast-bootstrap (Hoang et al. 2018). The tree was visualized using FigTree v1.4.4 (http://tree.bio.ed.ac.uk/software/figtree/).

## Supplementary Material

veac084_Supp
