# Supplementary material for: Monotreme-specific conserved putative proteins derived from retroviral reverse transcriptase
Source: Virus Evol. 2022 Sep 3;8(2):veac084. doi: 10.1093/ve/veac084 (PMC9514029; doi:10.1093/ve/veac084)
Supplement: veac084_Supp [file veac084_supp.zip › Supplemenrary_Figures.docx]

Supplementary Figure S1. (A) Distribution of bitscore obtained by the BLASTp search for platypus ERV-ORFs against echidna ERV-ORFs. The ORF pairs with high bitscore indicated by red lines were used for further analysis (Supplementary Table S2). (B) Schematic representation of the control screening for conserved ERV-derived genes in human and marmoset. (C) Distribution of bitscore obtained by BLASTp for human ERV-ORFs against marmoset ERV-ORFs. The numbers of hits with bitscore higher than 1500 are indicated by red arrows. The ORF pairs with high bitscore indicated by red lines were used for further analysis (Supplementary Table S6).

Supplementary Figure S2. Self-alignment of genomic nucleotide sequences around the RTOM genes in platypus and echidna. The nucleotide sequences were aligned using LAST (Kiełbasa et al. 2011) with “-E0.1 -C2 -m100” options. Alignments from simple repeats were removed using last-postmask. The dot plot was generated using last-dotplot (https://gitlab.com/mcfrith/last/-/blob/main/doc/last-dotplot.rst).

Supplementary Figure S3. Alignment of the whole GRIP2 region. (A) Platypus against echidna. (B) Platypus against human. (C) Platypus against human and mouse. (D) Echidna against human and mouse. Alignment and dot-plot were constructed using the LAST programs as described in Fig. S2.

Supplementary Figure S4. Comparison of the RefSeq transcripts and the assembled transcripts. (A) Gene coordinates of the RefSeq transcripts and the assembled transcripts. The main difference is that the RefSeq track does not contain echidna *RTOM1*. Another difference is that the assembled transcripts contain a chimeric transcript of *RTOM2* and *RTOM3* in echidna, which is transcribed from the *RTOM2* transcription start site and continues to *RTOM3* (red asterisk). (B) Comparison of RTOM transcripts obtained using unique-mapped reads and reads with ≤ 5 multiple mappings. To verify the possibility that the chimeric transcripts of *RTOM2* and *RTOM3* obtained by assembly are artifacts due to multi-mapped reads, we compared the coverage of unique-mapped reads with those of reads allowing 5 multiple mappings at maximum. As a result, the same splicing patterns and coverage were observed, suggesting that there is little concern about artifacts from multi-mapped reads. The RTOM ORFs were shown at the bottom of the coordinates.

Supplementary Figure S5. Screenshots of Integrative Genomic Viewer of RNA-seq reads on the *RTOM* genes. The transcript tracks in blue lines display the coordinates from the RefSeq GTF files. Thick blue lines indicate the coding sequences. Since there is no corresponding RefSeq transcript for echidna *RTOM1*, its gene coordinate was manually added from assembled transcripts in this study (Materials and Methods).


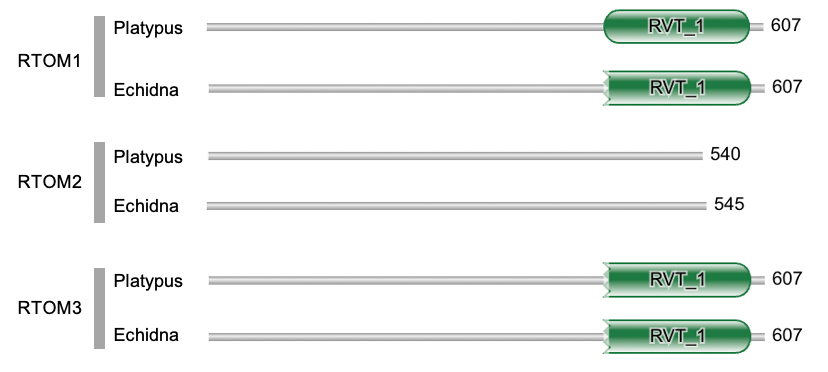


Supplementary Figure S6. The domains of the putative RTOM1, 2, and 3 proteins. The domain search was conducted using hmmscan in HMMER web server with default options (https://www.ebi.ac.uk/Tools/hmmer/search/hmmscan).


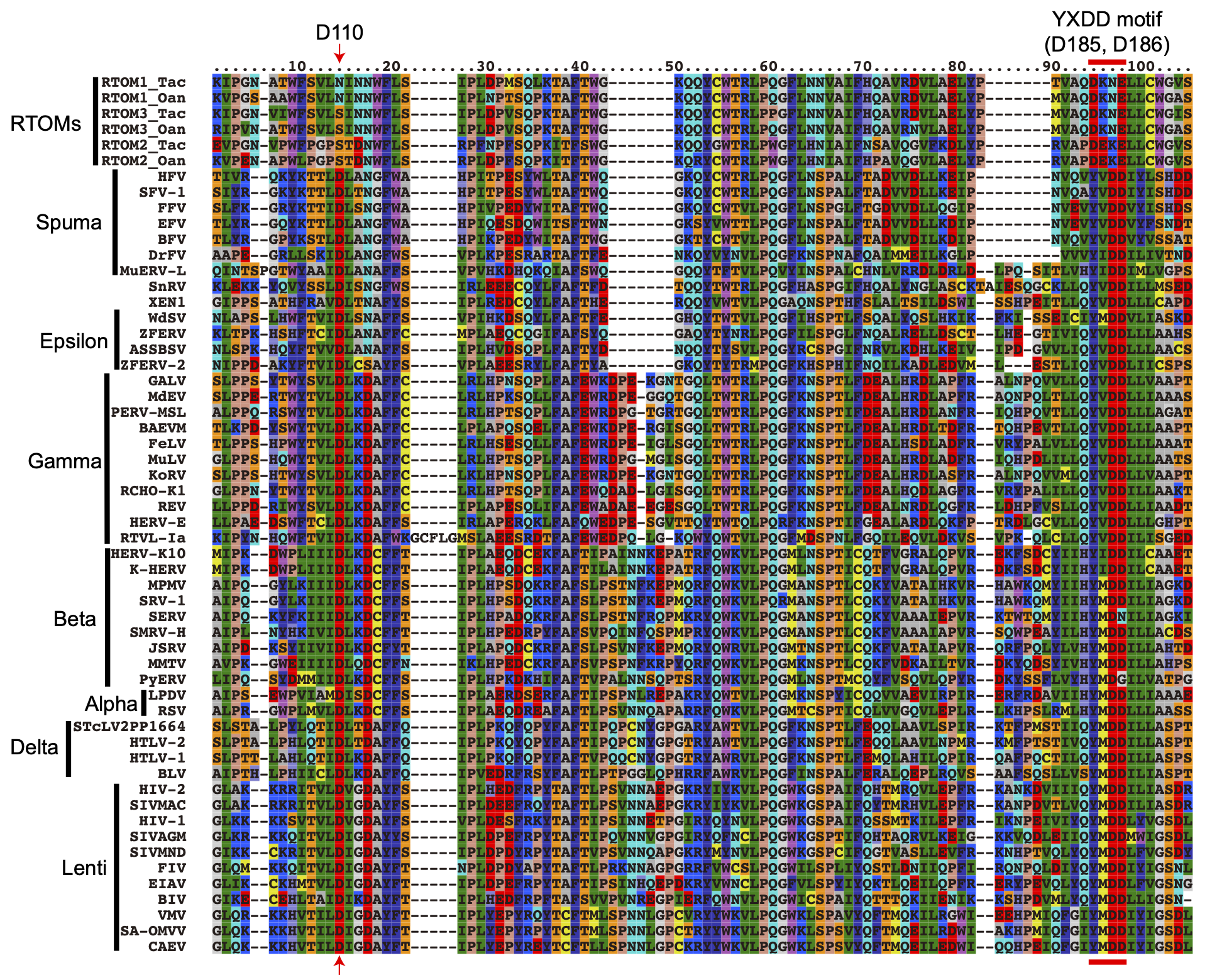


Supplementary Figure S7. Alignment of amino acid sequences of retroviral the RT region. The region corresponds to 513 - 614 amino acid sites of RTOM1_Tac. Three amino acids (D110, D185, and D186; locations based on the p66 subunit of HIV-1 RT) including the YXDD motif, which are the catalytic residues for reverse transcription and are highly conserved among all retroviruses, are shown in the alignment. Note that all RTOMs lack these amino acids.
