# Supplementary material for: Monotreme-specific conserved putative proteins derived from retroviral reverse transcriptase
Source: Virus Evol. 2022 Sep 3;8(2):veac084. doi: 10.1093/ve/veac084 (PMC9514029; doi:10.1093/ve/veac084)
Supplement: veac084_Supp [file veac084_supp.zip › Supplementary_file_1.docx]

**Supplementary file 1.** Nucleotide sequence of the echidna *RTOM1* transcript

>RTOM1_echidna_exon1:NC_052101.1:55487268-55487386:-

ggaaaaggcagttagtttggttttggccttgctgtggcatgtgcactgcctaacggctcccttgagacccagccagaagacatctccacattgttaaagtaactctctcagcacttagg

>RTOM1_echidna_exon2:NC_052101.1:55442464-55442598:-

gacataacagccccttagaaactgagtcacagagtgctactgacagaaatttcttcagccactggccccagccaaaaagactaagaaaatagagcctcaaatccagatacaaaccaagggactgctcacattcag

>RTOM1_echidna_exon3:NC_052101.1:55438460-55442187:-

tttcagggtcctggctggcccagatctggtactgcaagcttcagaactgaggaccaagaccggatttcatcagccagcaATGGAATCTCAGGAGCGGGAGATTGAAATCGAGTACCGCGTGGGGCAAACTTCTGGCAGGTTTTCATTGCGGCCCTCAAGACCATATACTCCCCCAAAGGCTAAGACATGGGTGGTTTTTAAGATTTGGGTGAGGGGTTCCCCCAATCCAGAGGGTAACAGCAAGCAGGATAAGGCTAAAGCAATTACCATGCAGGCTTTGGCCGAGGGCCTACATGATTTGTGGCGTAAACTGGATGGCAAGGACTTTTTGGTAGAAACCCTAACGTCTCACAATTTGTCCGAAGAGGATATGAATTTGACAGGGGAGAGGGTTGGAACCAGCAAAGGGTGGGGATATATAGTAACTAAGGAGTGGATAGTGGACCCCAATGGTCCCAGGATTCGGGACCCATACCTACTGCAATCTGCCTTACAGTCTTTAGTAGAGAGCATTCAAGATTTGTGGAATAGACTGGATGAAAAGGAGAAATCTAAGGAGCCAAGACCTGGGGACATTGTAGTAGAAAATGGATCATTTTCCAATGTGGTCCCACGTGACCTAATGAAATGGAAAGGGAAGAGGATGGGAGCCATCAGAGTCAGGGAAGAAGGAGAAACTACAGAGTGGAGAGTGGGGAGCAATTCCAACAGGATTAGAGGCCTATGGATGGAACTAGCTCCCTTTCAAGCTTTAAAAGTGGACACTCAGGATTGGTGGCATAGAGTGGTTGAAAATGAGCAAAATCCTTGGAATTCTGTAGTAGAAATGAGATTATTTAACAATGTGGGTAGAGCTGACCCAGTGAAATGGACAGGAGAGAGGGTAAGAGCAAACAGAGGTGGGGGAGATAGAGTTACGAGGGAGCAGAAAGTGGACACCAGTGCTCACAGGATTTGTGACCCAGAGGTGCAACAACTTGCCATCGACACAGCAGAGAGACCAAGCCTAGGAAGTAGGCCAGCCTCAAATGATTCCCCTTTAAAGCCTTGCTCCCTGCTGAAAATTTCCAAATACATGGCTATTAAGTCGCAGGAGGGTGCGGCAGAGGGAATGTTGAATTTTGAGCCACTGAAGGGTATCACCGAGGGAATTGTCCCACCAGGGTGGGAAGACACCTCCGAAGCCTGGGCCCGTGATAACCTCGATGCTGGCCAGTTGCAGGTGACCCCCATCATTATAGAAGGGGTGTTTCCCCCTAAACTCAAACAGTACCCCCTTCCTTTGGGGAGTATTGAGGAAGTGGTTAAGATGATACATATCTTGGAGAATCGTGGCTACATAAAGCCAAATATTTCACCCTCCAATGCTCCAGTGTGGCCAGTAAAGAAGCCCAGTGGCACGTGGAGCTTCCATATTGATTATAGGGCTTTGAACAGAGTGACATCTCCATTGACTCCCATGGTAACCACCTATCAAGATTTAGTGGATAAGATCCCAGGGAATGCGACCTGGTTCTCAGTACTGAACATTAACAATTGGTTTTTGAGCATACCGCTCGACCCCATGAGCCAGCTTAAGACAGCTTTTACTTGGGGGAAGCAGCAATACTGCTGGACTAGGCTGCCTCAGGGATTTCTTAACAATGTGGCCATTTTTCATCAAGCCGTGCGGGACGTTTTAGCAGAGCTCTACCCCACGGTGGCCCAAGATAAGAATGAGCTCCTCTGCTGGGGGGTTTCTAAGGAGGAGACCCAAAAGGCAACCAGGCTCATTATCCAGAGATTGAAAGATGCGGGCCTCAAGCTTGATGGCCATAAAGTTCAGTTGGTTCAAAGAGAAGTGTCCTTTTTAGGAATCAAGGTTGGGCCTTGTGGATGGAGGCTGGGCCCTATCAGTGTTTAAcaaattgaagccttgcaatccacctggaatcggttcaaccctttaaaggactggtagcggttataagagttctatccccatgccgctgccttacaagccttttatagaaattggttaaaacacagaagtttcacaggatggattgttataacaataatagtgtcattagtaaagggtttactaataggagaaatggagtctagcagagaagagtctgatggaagcccttttaaaggccccggtgtagtggaccccaaaagtgagtcttccaggcatcctttatggagaggaatggggcagggagaggcctcccatccttattccccgatagaatggacagtttgagaatgccaaaggggattattgggaatatacagggcctttcagaagcttaaacacctgactggagagtgtgatgtgactgtgtagacaccccacgtcaccctataatccacctgggccaagctgctccagggaatcactctggtctgaagaggcatccccaaagctgatatggtgggctttggtactatccaatccccagatctggtaccataaattaaatgtgatggatcgaggggctctgggtattctcttgagatttagaatccattactgtggagcaggttaatatgggtccgccattctatattccgccattcctgtgtggtgaatggacttcaaggatcagggaatgatctatgctttgcccaagaatcacaattaagaacggggctttaaaaggggaaaccatccttttcccccaggaatactagacaactatcgccacggttgttttcatggagatactacaatcccaacatcctgccccatccatatagccccttttgtttgggcagacctctttctggaatttatcttccctctcgactgcctgacctctgcacttgagtctgcacctttgtacccccgaagtactcacactacccctgcagcacttttataaatgtccttatattggattccttcacctactgtaatttattttaatgtccacctccctggctagagtgtaaaccccttgagagcagagattgtgcctacttattctgttgtgccacccaacctttctcaaccaatgccaacttgcttgttctcaggttctttcagtaattggattgtttgctcatttgcctgttttgatatataacaaataggaggaggcgagtattactgttgtactagtttggatattgatggaaacacaacaagccactcccaagtcaagccatccagttgtgcaagtgaacaaggttgtaattcatcccttctggccatcgactttaaccaggatccttcctaatggttttcctctgataaataata
