# Supplementary material for: Monotreme-specific conserved putative proteins derived from retroviral reverse transcriptase
Source: Virus Evol. 2022 Sep 3;8(2):veac084. doi: 10.1093/ve/veac084 (PMC9514029; doi:10.1093/ve/veac084)
Supplement: veac084_Supp [file veac084_supp.zip › Supplementary_file_2.docx]

**Supplementary file 2.** Nucleotide sequence of genomic ERV-ORF of RTOMs (from stop codon to stop codon). The methionine-started coding sequence on the RefSeq transcript (for platypus *RTOM1*, the assembled transcript in this study) is colored by red. Stop codons were colored by blue with underbar. Note that the gene coordinate of ORF is same as described in Table S2 and does not include stop codons.

>Ornithorhynchus_anatinus:NC_041749.1:50157125-50159155:- (RTOM1)

TAAAGTTATAAAAGTAAAGGTATTGAAATGTGTAGCTGTTCTTTCTACAACATTTGTGGGATGGGAATGGGTCAACAAAATCTATTTGTAGCTGTGTTAGGATGCTTGAGCTGCTTTTGTCTCCATAGTTTCAGGGTCCTGGCTGGCCTGGTAACAGATCTGGTACTGCAAGCTTCAGAACTGAGGACCAAGACCGGATTTCATCAGCCAGCAATGGAATCTCAGGAGCAGGAGATTGAAATCGAGTACCGCGTGGGGCAAAGTTTTGGCAGGTTTTCATTGCGGCCCTCAAGACCATATACTCGCCCAAAGGCTAAGACATGGGTGGTGTTTAAGATTTGGGTGAGGGGTTACCCCACTCCAGAGGGTGATAGCAGGCAGGATAAGGCTAAAGCAATTACCATGCAGGCTTTGGCCGAGGGCCTACATGATTTGTGGCGTAAATTGGATGGCAAGGACTTTTTGGTGGAAACCCTAACGTCTCACACTTTGTCCGAAGAGGATATGAATTTGACAGGGGAGAGGGGTGGAACTAGCAAAGGGTGGGGATATATAGTAACTAAGGAGTGGATAGAGGACCCCAGTGGTCCCAGGATTCGTGACCCAAATGTATTACAATCTGCCTTACAGTCTTTAGTAGAGAGCATTCAAGATTTGTGGAATAGACTAGATAAAGAGGAGAAATCCAAGAAGCCAAGTCCTGGGGACTTCATAGTAGAAAATGGATCATTTTCCAAAGTGATCCCACGTGACCTAATGAAATGGAAAGGGGAGAGGGTGGAAGCCAGCCAAGTGGGGGAAGAAGGAAGAACTAGAGAGTGGAGAGTGGGGAGCAATTCCAACAGGATTAGAGGCCTATGGATGGAACTAGCTCCCTTTCAGGCTTTAAAAATGGACACTCAGGATTGGTGGCATAGAGTGGTTGAAAATGAGCAAAGTCCTTGGAATTCTGTAGTAGAAATGAGATTATTTAACAGTGTTGGTAGAGCTGACCCCATGAAATGGACAGGGGAGAGGGTAGGAGCCAACAGAAGTGGGGGAGGTAGAGTTACGAGGGAGCAGAGAGTGGACACCAGTGCTCACAGGATTTATGACCCAGAGGTGCAACAACTTGCCGCCGACACAACGGAGAGACCAAGCCTAGGAAATAGGCCAGTCTCAAATGATTCCCCTTTAAAGCCACGCTCCCTGCTGGAAATTTCCAAATACATGGCTATTAAATCACTGGAGGGTGTGGCAGGGAGAATGTTGAATTTTGAACCACTGAACGGTATTACAGAGGGAATTGTCCCACCAGGGTGGGAAGACACCTCCGAAGCCTGGGCCCATGATAGTCTGGATGCTGGCCAGATGCAGGTGACCCCCATCCTTATAGAAGGGGCATTTCCCCCTAAGCTTAAACAGTACCCTCTTCCTTTAGGGAGTATTGAGGAAGTGGTTAAGATGATATATATCCTGGAGAATCGAGGATACATAAAGCCGAATATTTCACCCTCTAATGCTCCAGTGTGGCCAGTTAAGAAGCCTGATGGCACATGGAGCTTCAGTATTGATTATCGGGCCTTGAACAGAGTGACAGCTCCATTGACTCCAGTGGTAACCACCTATCAAGAGTTGGTGGATAAGGTCCCAGGGAGTGCGGCCTGGTTCTCAGTACTGAACATTAACAATTGGTTTTTGAGCATACCACTCAACCCCACGAGCCAGCCTAAGACAGCTTTTACTTGGGGGAAGCAGCAATACTGCTGGACTAGGCTGCCTCAGGGATTTCTTAACAATGTGGCCATTTTTCATCAAGCAGTGAGGGATGTTCTTGCAGAGCTCTACCCCATGGTGGCCCAAGATAAGAATGAGCTCCTCTGCTGGGGGGCTTCTGAGGAGGAGACCCAAAAGGTGACCAGGCTCATTATCCAGAAATTGACCGATGCGGGTCTCAAGCTTGATGGACATAAAGTTCAGTTGGTTCAAAGAGAAGTGTCCTTTTTAGGAATCAAGGTTGGGCCTTGTGGATGGAGGCTGGGCCCTATCAATGTTTAA

>Ornithorhynchus_anatinus:NC_041749.1:50135067-50136896:- (RTOM2)

TAAGTTTATAAAAGAAAAGATATTAAAGTCTGCACTTGCTCTTTCTGCCACATCGGCTGTCCAGGCGTGGGTCAACAAAGTCTACTTCTAGCTGTATCAGGATGCATGATCCGCTTTTGTCTCCAGAGTTTCAGGCTATTGGCTGGACCGGTAACAGATCTGGCCCTGCGAGCTTTGGAACTGAGGACCGAGACCGGATTTCATCAGCCAGCAATGGAATCTCAGGAGTCGGAATTTGAAATCGAGTACCTCGTGGGGCAAACTGCTGGCACCACCTCAGAGGAGCCGTCAGGATCTTATGCTCCCCCGGAGGATCAGGCATGGTTGGTGCTCAGGATTTGGGTGGAGGGTGCCCGTGACCTAGAGGGTAACAGCAAGCCGGATGAGGCTAAAGGGGTCGCCATACAGGCTTTAGCAGAGGGCCTGCATGATTTGTGGCGTAAACTGGAAGGCAAGGACTTTTGGATAGAAACCATATCATCTTACAATTTAGCTGAAGTGGATAGGAATTGGATGGAGAAGAGGGTGGGGCTCAGCAGAGGATGGGGCTACATCGTAAATAAGAGATGGAAAGTGGACCCCAATGGCCCCATTATCCGGGACCCACGTGTACTGCGGTCTGCCTTACAGACTCTAGTAGAGAGCATTCATGATCTGTGGTATCGACTGGGTCAAAGAGAAGATTCCAAGGATTCCCGTCCTTGGGAGTTTATCGTAGAAACCGGATCATTTGTCGATGAATTTCCGGATATCCCAATGAAAGGGGAAGGGGAGATGGAGGAAGAGAGTGGAGAGGAAGGAGACGAAGAATGGTGGGAAAAGGGAGCAGACACCAACGTACATGACATTTGGGGTCCGGGGGTGGAAGAGTCTGACATTGACACAGATGAGGAATCAAACCCAGAAGATGGGGCAGTCATGCATTATTCCCACTTCAAGGCACTTGCCCTCTGGGAGAGTCCCAAGTACATGACCGGTAAGAAGGGCATGGCACGGGGAATGTGGGATTTTGAGCCACTGAATGGTATCCTGCGGGAAATTGTCCCACCTGTGTGGGAAGACACCTCCGAAGCCTGGGCCCGTGATAGCCTGGAGGCTGACCAGCAGCAGGTGACCCCCTTCCATTTCCTCCCTAAGCATAAATGGTACCCCCTTCTTGAGGAGAGTAGTGAGGAAGACGTTCAGCCCATACACATCCTAGAGGATCGAGGTGATTTCATGCCAGGGGTGTCACCCTCTGATGCTCCACTGTGGCCAGCGAAGAAGTCGCGTGCCCCATGGAGCTCGAGGATTGACTCCTGGGCCTTGAGTGAGGCAACAGCCCAATTGATTCCAATGGTAACCACCTATCAAGGGTCAGAGGATAAGGTCCCAGAGAATGCGCCCTGGCTCCCGGGACCGAGCACTGACAATTGGTTTTTGAGCAGACCACTAGACCCCTTCAGCCAACCTAAGATAACCTTTACTTGGGGGAAGCAGCGATACTGCTGGACTAGGCTGCCTTGGGGGTTTCTTCATAACATAGCCATTTTTCATCCAGCAGTGCAGGGTGTTCTCGCAGAGCTCTACCCCAGGGTGGCCCCAGATGAGAAAGAGCTCCTCTGTTGGGGGGTTTCTGAGGAGAAGACACAAAGGGTGACCGGTATCATCATCAAGAGATTGACCGGTGTGGGCCTCAAGCTTGATGCACACGAGGCTCAGTTGGTCCAAAGAGAAGGGTCCTTTTTAGGCCTCAAGGTTGAGGCTTGTAAGTGGAAGCTGGGCCCTATCAGTCTGATCCTAATCAATCCAAACCTTGCCTCCTGCAACCTACCTGCAATCAGTCCAACCATGTAG

>Ornithorhynchus_anatinus:NC_041749.1:50120675-50122537:- (RTOM3)

TAAGCTTCAGGACTGAGGACCAAGACCAGATTTCATCATCCAGCAATGGCATCTCAGGAGCAGGAGTTCGAAGTGGAGTATCTCGTGGGGCAAACTGTTAGCAGCTGTTCAGTGATCAACTTAAGGCCGGATGATCCCCCGAAGGTTAAGACATGGGTAGTGGTTAAGGTGTGGGTGAAGGGTACCTGTGACCTAGAGGGTAACAGCAAGCAGGATAAGGCCAAAGCAACTGCCCTACAGGCTTTGGCAGAGGGCCTGCATGATTTGTGGCATACAGTGGATGGCAAGGACTTTATGGTAGAAACCGTTACCTCGTACAATTTGGATGAAGAGGATATGGATTTCATATGCAAGAGGGAGGGAGACAGTAGAGGGTGGGGATATAAAATAAGTAAGGAGTGGGTAGCAGACCCCAGTGGTCCCAGGATTCGTGACCCAAATGTATTACAATCTGCCTTACAGGGTTTGGTAGAGAGCATTCAAGATTTGTGGAACAGACTGGATGAAAAAGAGAAATTCAAGGAGCCAAGTCCTGGGGACTTTATAGTAGAAACCGGATCATTTTCCAATGTGGTCCCAGGTGACCTAATGAAATGGAAAGGGGAGAGGGTGGGATCCAGCCGAGTAGGGGAAGAAGGAAGAACTAGAGAGTGGAGAGTGGGGAGCAATTCCAACAGGATTAGAGGTTTAAAGGTGGATCTAGCTCCTTTTCAGGATTTAAAAGTGGACACTCAGGATTGGTGCCATAGACTGGCTGAAAATGAGCAAAATCCTTGGAATTCTGTATCAGAAATGGGATTATTTAACAGTGTGAGTAGAGCTGACCCAGTGAAATGGACAGGGGAGAGGGTAGGAGCCAACAGAGGTGGGGGAGATAGAGTTACAAGGGAGCAGATAGCAGACACCAATGCTCACAGGATTTGTGACCCAGAGGTGCAACAACTTGCCATTGACTTAGCAGAGAAACTAAACTTAGGAAATAGGCCAGCTGTAAAAGATTCCCCTTTAAAGCCAGGCTTCCCACTGAAATTTTCCCAAAACATGGCTACTAAGTCACTGGATGGTATGACAGGTGGAATGTTGAATTTTGAGCCACTGAAGGGTATCACAGAGGGAATTGTCCCACCAGGGTGGGAAGACACCTCCAAAGCCTGGGCCCGTGATGACCTGGATGTTGGCCAGATGCAGATGACCCCCATCATTATAGAAGGGGCGTTTCCCCCTAAACTTGAACAGTACCCCCTCCCTGTGGAGAGTATTGAGGAAATGGCTAATGTGATATACATCCTGAAGAATCGAGGTTACATAAAGCTAAATATTTCACCATCTAGTGCTCCACTGTGGCCAGTAAAGAAGCCCAATGGCACATGGAGCTTCAGTATTGATTACAGAGCCTTGAATAGGGTGACAGCTCGATTGACTCTAGTAGAAACCACCTATCAAGATTTAGTGGATAGGATCCCAGTGAATGCGACCTGGTTCTCCGTACTGAGCATTAACAATTGGTTTTTGAGCATACCGCTCGACCCTGTGAGCCAGCCTAAGACAGCTTTTACTTGGGGGAAGCAGCAGTACTGCTGGACTAGGCTGCCTCAAGGGTTTCTTAACAATGTGGCCATTTTTCATCAAGCAGTGCGGAATGTTCTTGCAGAGCTCTACCCCATGGTGGCCCAAGATAAGAATGAGCTCCTCTGCTGGGGGGCTTCTGAGGACGAGACCCAAAAGTTGACCAGGCTCATTATCCGGAGATTGAGAGATGCGGGCCTCAAGCTTGATGGACATAAAGTTCAGTTGGTTCAAAGAGAAGTGTCCTTTTTAGGAATCAGGGTTGGGTCTTTTAGATGGAGGCTGGGCCCTATCAATGTTTAA

>Tachyglossus_aculeatus:NC_052101.1:55440288-55442312:- (RTOM1)

TAAAGTGATAAAAGTAAAGGTATTGAAATGTGTAGCTGTTCTTTCTACAACATTTGCGGGATGGGAATGGGTCAACAAAATCTACTTGTAGCTGTATCGGGATGCTTGAGCTACTTTTGTCTCCATAGTTTCAGGGTCCTGGCTGGCCCAGATCTGGTACTGCAAGCTTCAGAACTGAGGACCAAGACCGGATTTCATCAGCCAGCAATGGAATCTCAGGAGCGGGAGATTGAAATCGAGTACCGCGTGGGGCAAACTTCTGGCAGGTTTTCATTGCGGCCCTCAAGACCATATACTCCCCCAAAGGCTAAGACATGGGTGGTTTTTAAGATTTGGGTGAGGGGTTCCCCCAATCCAGAGGGTAACAGCAAGCAGGATAAGGCTAAAGCAATTACCATGCAGGCTTTGGCCGAGGGCCTACATGATTTGTGGCGTAAACTGGATGGCAAGGACTTTTTGGTAGAAACCCTAACGTCTCACAATTTGTCCGAAGAGGATATGAATTTGACAGGGGAGAGGGTTGGAACCAGCAAAGGGTGGGGATATATAGTAACTAAGGAGTGGATAGTGGACCCCAATGGTCCCAGGATTCGGGACCCATACCTACTGCAATCTGCCTTACAGTCTTTAGTAGAGAGCATTCAAGATTTGTGGAATAGACTGGATGAAAAGGAGAAATCTAAGGAGCCAAGACCTGGGGACATTGTAGTAGAAAATGGATCATTTTCCAATGTGGTCCCACGTGACCTAATGAAATGGAAAGGGAAGAGGATGGGAGCCATCAGAGTCAGGGAAGAAGGAGAAACTACAGAGTGGAGAGTGGGGAGCAATTCCAACAGGATTAGAGGCCTATGGATGGAACTAGCTCCCTTTCAAGCTTTAAAAGTGGACACTCAGGATTGGTGGCATAGAGTGGTTGAAAATGAGCAAAATCCTTGGAATTCTGTAGTAGAAATGAGATTATTTAACAATGTGGGTAGAGCTGACCCAGTGAAATGGACAGGAGAGAGGGTAAGAGCAAACAGAGGTGGGGGAGATAGAGTTACGAGGGAGCAGAAAGTGGACACCAGTGCTCACAGGATTTGTGACCCAGAGGTGCAACAACTTGCCATCGACACAGCAGAGAGACCAAGCCTAGGAAGTAGGCCAGCCTCAAATGATTCCCCTTTAAAGCCTTGCTCCCTGCTGAAAATTTCCAAATACATGGCTATTAAGTCGCAGGAGGGTGCGGCAGAGGGAATGTTGAATTTTGAGCCACTGAAGGGTATCACCGAGGGAATTGTCCCACCAGGGTGGGAAGACACCTCCGAAGCCTGGGCCCGTGATAACCTCGATGCTGGCCAGTTGCAGGTGACCCCCATCATTATAGAAGGGGTGTTTCCCCCTAAACTCAAACAGTACCCCCTTCCTTTGGGGAGTATTGAGGAAGTGGTTAAGATGATACATATCTTGGAGAATCGTGGCTACATAAAGCCAAATATTTCACCCTCCAATGCTCCAGTGTGGCCAGTAAAGAAGCCCAGTGGCACGTGGAGCTTCCATATTGATTATAGGGCTTTGAACAGAGTGACATCTCCATTGACTCCCATGGTAACCACCTATCAAGATTTAGTGGATAAGATCCCAGGGAATGCGACCTGGTTCTCAGTACTGAACATTAACAATTGGTTTTTGAGCATACCGCTCGACCCCATGAGCCAGCTTAAGACAGCTTTTACTTGGGGGAAGCAGCAATACTGCTGGACTAGGCTGCCTCAGGGATTTCTTAACAATGTGGCCATTTTTCATCAAGCCGTGCGGGACGTTTTAGCAGAGCTCTACCCCACGGTGGCCCAAGATAAGAATGAGCTCCTCTGCTGGGGGGTTTCTAAGGAGGAGACCCAAAAGGCAACCAGGCTCATTATCCAGAGATTGAAAGATGCGGGCCTCAAGCTTGATGGCCATAAAGTTCAGTTGGTTCAAAGAGAAGTGTCCTTTTTAGGAATCAAGGTTGGGCCTTGTGGATGGAGGCTGGGCCCTATCAGTGTTTAA

>Tachyglossus_aculeatus:NC_052101.1:55424630-55426306:- (RTOM2)

TGAGCTTTGGAACTGAGAACCGAGACCAGATTTCATCAGCCAGCAATGGAATTTCAGGAGTCGGAATTTGAAATTGAGTACCTCGTGGGGCAAACTGCTGGCACCACCTCAGAGGAGCCATCAGGATCTTATGCTCCCCTGGAGGATCAGGCATGGTTGGTGCTCAGGATTTGGGTGGAGGGTGCACGTGACCTAGAGGGGAACAGCAAGCCGGATGAGGCTAAAGGAATCGCCATACAGGCTTTGGCAGAGGGCCTACATGATTTGTGGCGTAGACTGGAAGGCAATGACTTTTGGATAGAAACCATTTCATCTTATAATTTAGCTGAAGTGGATAGGAACTGGATGAAGGAGAGGGTGAGACTCAGCAGAGGATGGGGCTACATAGTAAATAAGAGATGGAAAATGGACCCCAATGGCCCCAAGATCCGGGACCCACATGTATTGCGGTCTGCCTTACAGACACTAGTTGAGAGCATTCACGATCTGTGGTATCAACTGGGTCAGAGAGAAGATTCCAAGGATCCCCGTCCTTGGGACTTTATCGTAGAAACTGGATCATTTGTTGATGAGTTCCCAGATATCCTGATGAATGGGGAAGGGGAGATGGAGGAAGACAGTGAAGACAGTGGAGAGGAAGGAGATGAAGAATGGTGGGAACAGGGAGCGGACACCGATGTTCACGACATTTGTGGTCCAGGGGTGGAAGATTCTGACATTGACACAGATGAGGAATCAATCCCAGAAGATGGGGCAGTCATGCATTATTCCCACTTCAAGGCATTCGCCCTCTGGAAGAATCCCAAGTATATGACCGTTAAGTCACTTAAGGGCATGGCAGGGGCAATGTGGGATTTTGAGCCACTGAATGGTATCCTGCGGGAAATTGTCCCACCTGTGTGGGAAGATACCTCTGAAACCTGGGCCCGTGATAGCCTGGAGGCTGGCCAGAAGCAGGTGACCCCCTTCCGTTCCTTCCCTAAGCATAAACTGTACCCCCTTCTGGTGGAGAGTAGTGAGGAAGAGGTTATGACCATACACATCCTGGAGGATCGAGGTGATTTCAGGCCAGGGATGTCGCCCTCTGATGCTCCACTGTGGCCAGTGAAGAAGTCATGTGGCCCATGGAGCTCGAGGATCGACTCCTGGGCCTTGAGTGAGGTGACAGCTCGATTGATTCCAATGGTAACCACCTATCAAGGGTCGGTGGATGAGGTCCCAGGGAATGTGCCCTGGTTCCCGGGACCGAGCACTGACAATTGGTTTTTGAGCAGACCGTTCAACCCCTTCAGCCAACCTAAGATAACTTTTTCGTGGGGGAAGCAGCAATACGGCTGGACTAGGCTGCCTTGGGGATTTCTTCATAACATAGCCATTTTTAATTCAGCAGTGCAGGGTGTTTTCAAAGACCTCTACCCCAGGGTGGCCCCAGATGAGAAAGAGCTCCTCTGTTGGGGGGTTTCTGAGGAGAAGACACAGAGGGTGACCAGTATTATCATCCAGAGATTGACCTGTGTGGGCCTCAAGCTTGATGCACGCAAGGCTCTGTTGGTCCAGAGAGAAGGGTCCTTTTTAGGCCTCAAGGTTGGGGCTCGTAAGTGGAAGCTGGGCCCTATCAGTCTGATCATAATGGATCCAAACCTTGCCTCCTGCAACCTACCTGCAATCGGTCCAACCGTGTAG

>Tachyglossus_aculeatus:NC_052101.1:55408366-55410228:- (RTOM3)

TAAGCTTCAGAACTGAGGACCAACACCAGATTTCATCAGACAGCAATGGAACCTCAGGAGCAGGAGTTCGAAATGGAGTACTGTGTGGGGCAAACTGTTGACAGCTGTTCAGTGATCAACTTAAGGCCGGATGATCCTCCGAAGGTTAAGACATGGGTAGTGGTTAAGGTGTGGGTGAAGGGTACCCGTGACCTAGAGGGTAACAGCAAGCAGGATAAGGCCAAAGCAGCTGCCCTACAGGCTTTGGCAGAGGGCCTGCATGATTTGTGGCGCACACTGGATGACAAGGACTTTGTGGTAGAAACCATTACATCATACATTTTGGCTGAAGAGGATATGGATTTCATAGGCAAGAGGTGGGGAGACAGTAGGGGGTGGGGATATAAAATAAGTAAGCAGTGGGTAGCAGACCCCAGTGGTCCCAGGATTCGTGACCCAAACGTATTACAATCTGCCTTACAGGGTTTGGTAGACAACATGCAAGATTTGTGGAATAGACTGGATGAAAAAGAGAAATTCAAGGAGCCAAGTCCTAGGGACATTATAGTTGAAAGCAGATCATTTTCCAATTTGGTCCCAGGTGACCTAATGAAATGGAAAGAGGAGAAGGTAGGAGCCAGCAGAGTGGGGGAAGAAGGAGAAACTAGAGAGTGGAGAGTGAGGAGCAATTCCCACAGGATTAGAGGCCTAAAAATGGCTCTAGTTCCCTTTCAGGCTTTAGAAGTGGACACTCAGGATTGGTGCCATATACTGGCTGAAAATGGGCAAAATCCTTGGAATTCTGTATTAGAAATGGGATTATTTCACAGTGTCAGTAGAGCTGACCCAATGAAACGGACAGGGGAGAGGGTAAGAGCCAACAGAGGTGGGGGAGATAGAGTTACGAGGGAGCAGACAGTGGACACCAATGCTCACAGTATTTGTAACCCAGAGATGCAACAACTTGCCATTGACACAGCGGAGAGACTAAGCCTAGGAAATAGGCCAGTTGTAAAAGATTCCCCTTTGAAGCCATGCTTCCCACCGAAAATTTCCAAATACATGGCTATTAAGTCGTTGGAGGGTGTGGCAGGGGGAATGTTGAAATTTGAGCCACTAATGGGTATCACCAAGGGAATTGTCCCATCAGGGTGGGAAGACACCTCCGAAGCCTGGGCCCGTGATAACCTGGATGTTGGCCAGATGCAGGTGACCCCCATCATTATAGAAGGGGCGTTTCCTCCTAAACTTAAACAGTTCCCCCTCCCTTTGGAGAGTATTGAGGAAATGACTAAGACGATATACATCTTGAAGAATCGAGGATACATAAAGCCAAATATTTCACCATCTAGTGCTCCACTGTGTCCAGTAAAGAAGCCTGATGGCACGTGGAGTCTCAATATTGATTATAGGGCCTTGAATAGAGTGACGGCTCGATTGAGTCTAGTAGAAACCACCTATCAAGATTTAGTGGATAAGATCCCAGGGAATGTGATCTGGTTCTCTGTACTGAGCATTAACAATTGGTTTTTGAGTATACCGCTCGACCCTGTGAGCCAGCCTAAAACAGCTTTTACTTGGGGGAAGCAGCAATACTGCTGGACTCGGCTGCCTCCAGGGTTTCTTAACAATGTGGCCATTTTTCATCAAGCAGTGCGGGATGTTCTCGCAGAGCTCTACCCCATGGTGGCCCAAGATAAGAATGAGCTCCTCTGCTGGGGGATTTCAGAGGAGGAGACCCGAAAGTTGACCAGGCTCATTATCCAGAGATTGAGAGATGTGGGCCTCAAGCTTGATGGACATAAAGTTCAGTTGGTTCAAAGAGAAGTGTCCTTTTTAGGAATCAGGGTTGGGCCTTGTAGATGGAGGCTGGGCCCTATCAATGTTTAA
